# Is There a Convergence between the Food Classification Adopted by Food-Based Dietary Guidelines and Food Science and Technology?

**DOI:** 10.3390/foods12203824

**Published:** 2023-10-18

**Authors:** Jordanna Santos Monteiro, Raquel Braz Assunção Botelho, Renata Puppin Zandonadi, Wilma Maria Coelho Araujo

**Affiliations:** Department of Nutrition, School of Health Sciences, University of Brasília, Campus Darcy Ribeiro, Asa Norte, Brasilia 70910-900, DF, Brazil; jordanna.santosmonteiro@gmail.com (J.S.M.); raquelbotelho@unb.br (R.B.A.B.); renatapz@unb.br (R.P.Z.)

**Keywords:** food classification, food-based dietary guidelines, food science and technology

## Abstract

The World Health Organization (WHO) proposed the dietary guidelines presented as the Food-based Dietary Guidelines (FBDG). The FBDG classify foods according to their origin, nature, nutrient source, food group, and processing level. Food science and technology (FST) ranks food according to its origin, perishability, nutrient source, processing, food group, and formulation. This paper aimed to compare the convergence points for food classification according to the FBDG and FST. This study was carried out in two phases. The first step was identifying the Food-Based Dietary Guidelines (FBDG). For each of the FBDG, food items were grouped as fruits, vegetables, cereals, sugars, fat and oils, legumes, foods from animals, dairy products, and others. The second step aimed to identify and describe the different food classification systems. The search was performed on PubMed^®^, Science Direct, and Web of Science and websites of international organizations such as the Food and Agriculture Organization of the United Nations (FAO), the World Health Organization (WHO), and the *Codex Alimentarius*. Our results show that the points of convergence between the classifications were the classification in terms of origin (animal and vegetable), nutrient sources, and food groups. However, inconsistencies were observed for the distribution of food items in each group in the 98 surveyed FBDG. As for nature, there was a convergence for in natura, minimally processed, and processed foods. However, the criteria adopted for minimally processed and processed foods described in the FBDG differ from those considered by the FST. FST also does not recognize the classification of foods concerning the level of processing.

## 1. Introduction

Food consumption has been recognized as an essential predictor of health or behavior that strongly influences health and future disease risk. Food composition and classification data are used in health studies, from which results are the basis for formulating public policies [1,2,3,4,5,6]. However, in epidemiological studies, comparing food and nutrient consumption in different countries indicates difficulties in finding a solid foundation for classifying food [7,8,9,10,11,12]. 

Such divergences are probably observed because two fields of scientific knowledge, although closely related, assign different criteria to classify foods: the first referring to food production and the second related to food and its effects on health. Many sciences, such as physics, chemistry, mathematics, and biology, bypassed the scientific development of food science and technology [13,14]. At the beginning of the 19th century, dietary standards emerged to regulate the production and marketing of food products and ensure consumers’ health and legal commercial practices. In this scenario, in 1954, the Codex Alimentarius Europaeus was established, comprising all European countries. In 1961, the Codex Alimentarius Commission was established at the 11th Food and Agriculture Organization (FAO) Conference. In 1963, the World Health Assembly adopted the Joint FAO/WHO Food Standards Program. Thus, the Codex Alimentarius has become a world reference point for consumers, food producers, and processors for international food control and trade bodies. Its influence extends to all continents, and its contribution to protecting consumers’ health and ensuring fair practices in the food trade is incalculable [15,16,17,18]. 

The Codex Alimentarius covers topics related to food production at all stages of the production chain. It includes processed, semi-processed, and in natura foods. It also addresses issues related to the production of conventional and specialty foods, labeling, food hygiene, food additives, pesticide residues, and food safety assessment procedures derived from modern biotechnology, food inspection, and certification [15,16,17]. 

The changes from the First and Second World Wars in the production and sale of food, resulting from the economic, political, and social context, also led the population to move from a situation of malnutrition prevalence to the current state, in which excess weight takes on epidemic proportions [19,20,21,22,23]. In this context, another scientific field enters the scene with its epidemiological studies, which primarily require the establishment of food classification or even the elaboration of a classification system that allows the evaluation of the impact of food on human health [3,12,24,25].

In this sense, the proposals for organizing, creating classification systems, food description systems, or even categories for describing foods may have different objectives, contributing to generating other groups: to create databases on the composition of foods; to support research and technological development; to monitor international, national, and regional trade in food items; to study the relationship between food consumption and health; to monitor the use of pesticides, dyes, antibiotic residues, and veterinary drugs, among others [8,26,27,28,29].

The classification of foods aims to group them according to their physical, chemical, nutritional, and biological characteristics, as well as other food components, seeking, among other interests, the creation of programs and policies in the area of nutrition, health, and in the fields of agriculture and food industry [30,31,32,33]. Food classification is a list of different foods in groups, with or without subgroups, based on common properties and mainly identified by the consumer and food professionals. This grouping identifies a collection of food items that are not generally considered variants of the same food but share essential characteristics regarding nature, origin, or use [10,14,34]. Table A1 (Appendix A) describes some of the food classification systems developed for carrying out epidemiological studies: the Data Food Networking (DAFNE); the European Prospective Investigation into Cancer and Nutrition (EPIC); the Euro Food Groups (EFG); the Food Classification and Description System (FoodEx); NOVA system and others [5,10,14,25,28,35,36]. 

Food classification systems aim to organize and distribute a set of elements according to an order established by legal documents, consumers, and food professionals [8,10,14]. Some examples are The Langual Thesaurus, which is organized into 14 facets of food’s nutritional and hygienic quality, and the INFOODS Guidelines for Describing Foods, proposed by the INFOODS Food Nomenclature and Terminology Committee [8]. A food category is a term that identifies a set of food items that share generic characteristics, for example, in terms of nature or use as the category of grains and derivatives or alcoholic beverages [10].

Classifying food in multiple dimensions (including culturally recognized and socially significant ones) is possible. However, it is essential that a food classification standard becomes a reference for the population and data obtained in epidemiological research in different regions of the world are comparable and equally interpreted, and understood by consumers, professionals, and scientists in the public health, nutrition and FST [37,38,39]. In this sense, food classification should be understandable for consumers and specially designed for those who work with this professionally. There is consensus on the need for a harmonious, globally accepted, and implemented food nomenclature and classification. Data on the chemical composition of foods must express reliable information about the nutritional composition of foods [8,26]. 

In this regard, in 1998, the World Health Organization (WHO) proposed dietary guidelines as Food-based Dietary Guidelines (FBDG). FBDG aim to guide the population regarding food consumption and recommend that meal planning is based on foods that provide a healthy and balanced diet. They incorporate the consumption characteristics of each country and their eating habits, providing recommendations for which foods to eat or not [2,34,40,41,42]. 

The FBDG classify food items according to their origin, food groups, nature/processing, and nutrient sources and food science and technology (FST) classifies foods according to the degree of perishability, origin, nutrient source, nature, processing, and formulation [8,10,12,43,44,45,46,47]. Considering that the FDBG are essential tools for promoting healthy eating, that a critical number of studies on food consumption are carried out and have as standard the classification of foods described in such documents, there is a gap in the literature on research comparing the food classification proposed in the FBDG and by FST, this paper aimed to compare the convergences between the two types of food classification.

## 2. Methodology 

**Figure 1:**
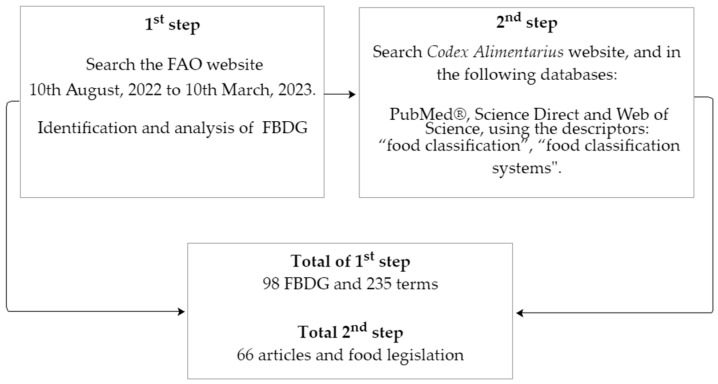
Phases of Food-based Dietary Guidelines (FBDG) analysis and search.

This study was carried out in two phases (Figure 1). The first step was identifying the Food-Based Dietary Guidelines (FBDG) on the FAO website [43] from 10 August 2022 to 10 March 2023. For each of the FBDG, food items were grouped as fruits, vegetables, cereals, sugars, fat and oils, legumes, foods from animals, dairy products, and others. To organize FBDG data, a database was created using “Microsoft Excel” (2016) software (Office Windows package). Data were organized into three software files using “Microsoft Excel” (2016). The countries’ languages were registered in the first file and the first worksheet. In the second worksheet, the names of all the food classifications of all the FBDG were included. Based on the obtained classifications, the food groups were separated by spreadsheets. The presence or absence of a particular food in a certain group was organized in a second file in the software “Microsoft Excel” (2016). For example, green banana was included in the cereal or fruit group. The third file presented all the classifications for each food group: all the nomenclatures that only refer to cereals or carbohydrate sources in a spreadsheet, all nomenclatures that only refer to meat or protein sources in another spreadsheet, and so on. Finally, all the food groups on the FAO website were systematized. The data for each worksheet were calculated using the “count” or “sum” feature and the percentage value of the groups or foods.

The second step aimed to identify and describe this study’s different food classification systems. The search was performed on PubMed^®^, Science Direct, and Web of Science and websites of international organizations such as the Food and Agriculture Organization of the United Nations (FAO), the World Health Organization (WHO), and the *Codex Alimentarius*. The following combinations of descriptors were used to search: “food classification” and “food classification systems.” The search was performed on 10 August and 11 August 2022, without limitation on the date or origin of the studies. To locate possible studies not found in the initial search, the authors performed a reverse search using the reference lists of the selected articles. Inclusion criteria were original and review articles on (1) food classification systems, (2) food guides, (3) food processing, (4) food industry, (5) industrialized food, and (6) processed food. Exclusion criteria were randomized clinical trials, experimental studies, case studies, and studies that brought an association between a particular food group and/or dietary guidelines and diseases. A total of 66 articles and international documents (specific legislation) were included (Figure 1). 

## 3. Results

### 3.1. Food Classification According to the Food Science and Technology (FST)

**Figure 2:**
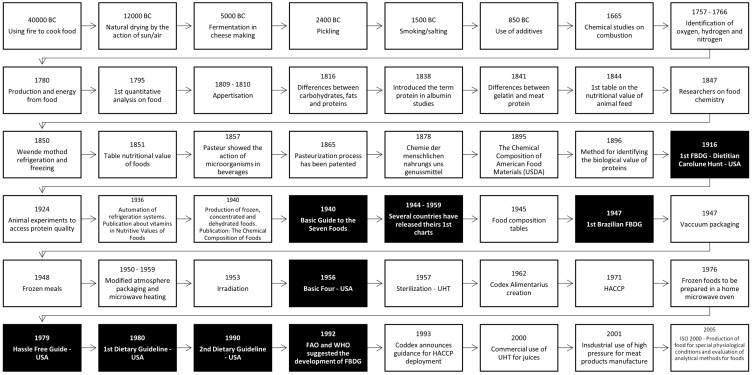
Evolution of food science and technology (white) and Food-based Dietary Guidelines-FBDG (black).

Processed foods have been part of our diet since ancient times. From making bread in 25,000 BC, foods such as dried fruits, olive oil, cheeses, preserves, chocolate, bacon, salted and cured meats, and sugar were obtained through artisanal techniques [48,49,50,51,52,53]. Studies performed between the 17th and 19th centuries led to the identification of chemical and biological events such as combustion; the naming of elements (oxygen, hydrogen, and nitrogen); the performance of the first chemical analyses; the identification of food components such as proteins; the confirmation of Pasteur on the action of microorganisms in alcoholic beverages; the pasteurization process; the production of the first preserves, consolidating technologies as a science (Figure 2). Scientists such as Lavoisier, Gay-Lussac, and Berzelius contributed to modern food chemistry, initially stimulated by the knowledge of food composition [48,49,50,51,52,53,54,55,56,57]. 

The evolution of food science from the 17th century provided the opportunity to evolve from artisanal techniques to contemporary technologies. The need to achieve and maintain food safety and the interest in extending seasonal foods’ shelf life led to food-processing developments. Incorporating scientific principles formed the basis of the processes currently used to manufacture food products and ingredients. We have evolved towards optimizing technologies, standardization of products, increasing the offer, and reducing the occurrence of physical, chemical, and biological risks in food [48,49,50,51,52,53,58]. Additionally, in the last three centuries, in addition to improving analytical techniques, science has shown that several factors influence the variability in nutrient content (varieties, species, climatic conditions, type of production, place of production, and processes) [38,49,59]. 

Processing is related to the transformations that the food undergoes, such as washing, grinding, mixing, cooling, storing, heating, freezing, filtering, fermenting, extracting, extruding, centrifuging, frying, drying, concentrating, pressurizing, irradiating, microwaving, packaging, and preservation needs in agricultural societies to prevent postharvest losses. Today, food processing enables the maintenance of international commerce, assuring safe, palatable, and nutritionally adequate products, besides reducing food losses, which is strategic for food security [36,50,60,61,62,63,64,65,66]. 

Any process, or even any continuous sequence of operations, is defined as a step, among many, that leads to the transformation of fresh food. Each step refers to an essential operation that results in physical and chemical changes in the food. Processing may also include adding other ingredients such as salt, sugar, food additives, and other substances approved in formulations. Therefore, processing can reduce, increase, or maintain unaltered properties of in natura foods or even minimally processed foods [36,58,61,67,68,69].

**Figure 3:**
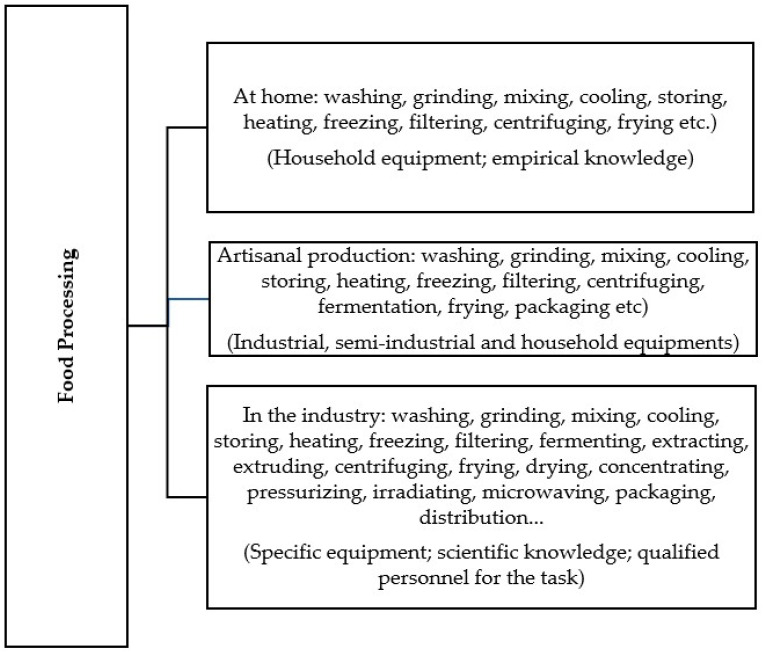
Types of operations used in food processing in the household environment, in artisanal production, and in industry.

It is essential to understand that there is a difference between processed and industrialized food, despite the terms being mistakenly used as synonyms. Industrialized food can also be classified as processed food. Industrialized foods are processed using equipment appropriate to the production volume in their facilities. However, the opposite is only sometimes true, as food processing can also be carried out in homes and various food services, such as cafeterias, restaurants, schools, hospitals, or even farms, among many others [36,69,70]. Artisanal food production, for commercial purposes (or not), whether on a small or large scale, is also an industrial activity, as the food is processed to be offered to a population and not for their consumption (Figure 3) [69,70].

The classification criteria supported by FST are based on similar characteristics among different foods, such as origin, nutrient source, perishability, nature, processing, and formulation (Table 1) [26,45,59,60,70,71]. As for origin, foods are classified as species of plant and animal origin. Foods are dietary sources of proteins, carbohydrates, lipids, vitamins, and minerals. For the Codex Alimentarius, vegetables are plants cultivated in the field and garden crops in the open and under glass. They comprise all plant species consumed as food obtained directly from the soil, without any transformation other than cleaning, maintaining all the biological qualities they had when they were still in the plants [59]. Vegetables are grouped according to botanic characteristics as follows: leafy or stem vegetables and flowers (e.g., cabbage, lettuce, and arugula); fruit-bearing vegetables (e.g., melons, tomatoes, and peppers); root, bulb, and tuberous vegetables (e.g., onion, potatoes, and sweet potatoes); leguminous vegetables (e.g., green peas); other vegetables (e.g., green maize and mushrooms). Fruit is the organ that comes from the flower, formed by the maturation of one or more ovaries after fertilization. They are generally classified as simple, aggregated, and multiple (or compound) fruits.

Vegetable classification can also be based on climate adaptation (hot climate and cold climate); the life cycle (annual, biannual, perennial); origin (exotic and autochthonous); the way the product is presented to the consumer (in natura, canned, dehydrated, frozen, minimally processed, and processed); based on the edible parts (roots, tubers, rhizomes, leaves, fruits, tender stalks and buds, inflorescences/flowers, bulbs, immature seeds, and cultivated mushrooms). The botanical characteristic is the most appropriate for naming these food items in their groups because they are stable. For this purpose, three taxonomic units are used: the botanical family (grouping of related botanical genera), the botanical genus (group of related species), and the botanical species (basic taxonomic unit), bringing together very similar plant characteristics [72,73,74,75,76,77]. 

**Table 1:** Food classification according to food science and technology.

Food Classification		Reference
**Origin**	Products of animal and vegetable origin	[59]
**Nutrients Sources**	Sources of proteins, carbohydrates, lipids, vitamins, and minerals	[59,60]
**Perishability**	Perishable (A_w_ value ≥ 0.85) Semi-perishables (A_w_ value ≤ 0.85)	[60,71,78]
**Nature**	In natura, minimally processed, and processed food	[70]
**Processing**	Processing at room temperature: cleaning, sorting, peeling, size reduction, mixing, shaping, separation, concentration of components, fermentation, and addition of enzymes	[61,70,79]
	Processing by application of heat: bleaching, pasteurization, sterilization, evaporation, extrusion, dehydration, dielectric heating, ohmic, and infrared	
	Heat removal processing: controlled or modified atmosphere cooling, storage and packaging, freezing, lyophilization, and freeze concentration	
	Processing by application of irradiation, electric fields, high hydrostatic pressure, and light or ultrasound	
**Formulation**	Simple and mixed foods	[45]

Cereals are vegetables that, botanically, belong to the grass family. Their seeds are harvested when dried and comprise species such as rice, wheat, corn, barley, triticale, rye, millet, sorghum, and oats [77,80]. Cereal-based products are obtained from edible parts that can be subjected to maceration, milling, extraction, heat treatment, and/or other technological processes considered safe for food production, such as bran, starchy products, bread flour, pastry, pasta, and beverages (beer), among others [80]. Legumes (pulses) are protein-containing and mainly consumed when dried, but for some species, legumes can also be consumed in the immature phase. Bean (*Phaseolus vulgaris*), broad bean (*Vicia faba*), lentil (*Lens culinaris*), chickpea (*Cicer arietinum*), dry pea (*Pisum sativum*), and vigna (*Vigna* sp.) are considered “pulses”, and they are distinguished from leguminous oil seeds by their low-fat content [77,81,82,83]. 

Oilseed consists of seeds from various plants that produce edible vegetable oils, seed meals, and cakes for animal feed. Some important vegetable oil seeds are by-products of fiber or fruit crops (e.g., cottonseed and olives). Some of the oilseeds are, directly or after slight processing (e.g., roasting), used as food (e.g., peanuts) or for food flavoring (e.g., poppy seed and sesame seed) [17,59]. 

According to *Codex Alimentarius*, meat is the matured muscle mass and the other tissues accompanying it, including the corresponding bone mass, offal, blood, fat, cartilage, and bones. Its classification is related to the species from which it precedes: beef, pork, lamb; poultry (chicken, turkey, and duck); fish (fish, lobster, shrimp, and oysters); hunting (non-domestic animals); reptiles; batrachians (e.g., frogs and others); chelonians (e.g., turtles and others); insects [59,84]. In turn, the eggs of some species of animals (birds or reptiles) are fertilized, or not. The designation of egg means chicken eggs; the others come with an indication of the species. In contrast, seafood means fish, crustaceans, mollusks, amphibians, reptiles, echinoderms, and other aquatic animals used in human food [85,86,87]. Milk without other specifications is derived from complete and uninterrupted milking under hygienic conditions from healthy, well-fed, and rested cows. Dairy products are obtained through the technological processing of milk and may contain ingredients, additives, and technical aids only when functionally necessary for processing [86,88]. 

Foods of animal and vegetable origin can be classified as perishable or semi-perishable since the water activity content in the food matrix affects the speed of deterioration, mainly of microbial origin. They can be classified as in natura, minimally processed, and processed according to their nature. Fresh food refers to food in its natural state [36]. Minimally processed food is any product, usually vegetables, e.g., roots and tubers, leaves, fruits and flowers, pods and seeds, sprouts, and fruits, or any combination thereof, that has been physically altered from its original form and remains fresh. In turn, processed food is the raw material (food item) of animal or vegetable origin, which has been subjected to processes that can occur at room temperature, by the application of heat, by removal of heat, by irradiation, electric fields, high hydrostatic pressure, and light or ultrasound. It is also possible to use food additives or add nutrients or bioactive substances [36,60,70,89,90]. 

In this context, foods are classified as pasteurized, dehydrated, evaporated, extruded, refrigerated, frozen, lyophilized, irradiated, fermented, and enriched, among others [45,61,70,91,92]. However, the chemical composition and, consequently, the nutritional value of such foods will also depend on preliminary operations such as peeling, size reduction, mixing, shaping, separation, the concentration of components, etc.; therefore, we infer that the nutritional value, for example, of brown rice (richer in fiber) and processed rice (richer in starch) differs from the nutritional value of the grain in natura [45,91]. In the same way, it is necessary to pay attention to the formulation of the products. Formulation is the term used in the food industry to describe the amount of ingredients and food additives calculated from the amount of raw material, which corresponds to 100%. The formulation makes it possible to predict which technologies (processes) are necessary to guarantee the quality of the products in terms of chemical, microbiological, physical, sensory, nutritional, and legal aspects [36,93].

Thus, the International Network of Food Data Systems (INFOODS) proposes the classification of foods based on their composition as simple foods and compound foods. Simple foods are those that have the following criteria: (a) foods in their natural state, only inedible or rejected parts are removed (fruit pulp, fruits, and vegetables in natura); (b) foods from which part of the edible portion has been removed during processing (skimmed milk, white wheat flour); (c) foods with a single main ingredient, dehydrated or with added water (dried fruits, cooked rice, teas, fruit juices, concentrated or diluted, fruit nectars); (d) foods with a single main ingredient, added with other ingredients in amounts that do not significantly impact the energy value; (e) foods that have been processed with or without the removal of parts of the edible portion, with or without the addition of small amounts of other ingredients, such as fortified corn flakes. Compound foods consist of raw materials with ingredients from different sources, such as cakes, bread, and ready-to-eat products, among others [10,28,45,94]

### 3.2. Food Classification according to the Food-Based Dietary Guidelines (FBDG)

Contrary to the expectations around adopting FBDG by different countries that make up the United Nations, of the 193 countries, only 51% (*n* = 98) have FBDG to guide the consumption of necessary foods for health promotion. The regions with the highest number of FBDG are the European continent (*n* = 34), the American continent—Latin America and Caribbean (*n* = 29), and the Asian continent—Asia and Pacific (*n* = 17). Although Africa has 54 countries, only 10 have FBDG; Oceania has 6 countries with FBDG (Table 2). 

The most usual systematization in the FBDG is food groups (*n* = 94; 96%). Although 94 FBDG use classification according to food groups, we identified that some countries adopt combinations of food groups with nature and/or nutrient sources and/or origin, such as the FBDG of Peru, Israel, and Belgium, among others. Alternatives such as nutrient sources (carbohydrates, proteins, lipids, vitamins, and minerals) (*n* = 13, 13%) or according to their nature/processing (*in natura*, minimally processed, and processed) (*n* = 10, 10%), origin (vegetable and animal) (*n* = 17, 17.5%) and culture of the country are the minority (*n* = 2, 2%). 

In seven FBDG (from Yugoslavia, Slovenia, Croatia, Iceland, Ecuador, Peru, and Brazil), foods are classified as highly processed foods (*n* = 1); highly processed foods, rich in sugar and fat (*n* = 1); processed foods (*n* = 5); processed foods high in fat, sugar, and salt (*n* = 1); in natura or minimally processed (*n* = 3); ultra-processed foods (*n* = 4). Of these countries, Brazil, Ecuador, and Peru use the NOVA classification (in natura or minimally processed foods; oils, fats, salt, and sugar; processed foods and ultra-processed foods) [12,35,95]. Brazil and Ecuador are the only ones that adopt the classification according to nature/processing, while Peru also adopts the classification of food groups. Sweden and Fiji classify food based on healthy parameters. 

**Figure 4:**
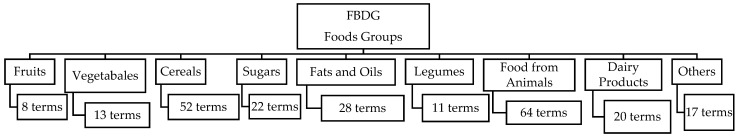
Terms used to describe food items in the respective food groups cited in the 98 FBDG.

Two hundred thirty-five terms were cited to name the food items in the respective groups described in the 98 FBDG (Figure 4). The most-used terms to identify foods in the 98 FBDG were: fruits (*n* = 57; 58%), vegetables (*n* = 52; 53%), oils and fats (*n* = 27; 27.5%), vegetables and fruits (*n* = 24; 24.5%), and foods from animals (*n* = 13; 13%).

It is important to mention that the “dairy products” group is separated from the “Food from animals” group because, although the name food from animals is the most used for foods of animal origin, not all food groups of animal origin include dairy products (there are 30 terms related to the specific nomenclature “milk or dairy products”). Therefore, this separation was maintained in this manuscript.

### 3.3. Food Group Analysis 

#### 3.3.1. Fruits, Vegetables, Cereals, and Legumes 

We found 84 terms to specify food items in the fruits, vegetables, cereals, and legumes groups. In total, 8 terms categorized foods into the fruit group (*n* = 8; 8% of the 98 FBDG), 13 for the vegetable group (*n* = 13; 13% of the 98 FBDG), 52 (53%) for the cereal group, and 11 (11.2%) terms were listed as foods belonging to the legumes group (Figure 4; Appendix B, Table A2). We also identified in the 98 FBDG that 47% (*n* = 46) described fruits and vegetables in the same group; 20% (*n* = 20) included fruit juice; nine (10%) included beans; in eight (8%) FBDG, legumes were also part of this group. Forty-seven FBDGs included legumes in the protein sources group [83,96,97,98,99].

It is plausible to consider that for the construction of these groups, the inclusion of items with common properties and easily identifiable by the consumer was considered. However, our study identified that the items listed by the group have widely differing internal and external characteristics. They differ regarding the relevant data set to the grouping, such as shape, colors, consumption and preparation characteristics (process), chemical composition, and nutritional value. Only their origin, e.g., plant origin, is the common criterion.

#### 3.3.2. Food from Animals

Likewise, we identified that some of the terms used to name the food items belonging to the legumes group in the analyzed 98 FBDG are related to foods belonging to other groups, such as nuts (fruit), soya (oilseed), and meat (food from animals). Of the 235 identified terms, 27% of these (*n* = 64) were foods from animals or protein sources, such as meat, poultry, pork, game meat, offal, fish, seafood, meat products (sausage, bologna, salami, bacon or ham), insects, eggs, tofu, milk, yogurt, cheese, beans, soy, pulses, peanuts, seeds, and oilseeds/nuts (Figure 4; Appendix B, Table A2; Appendix C, Table A3). We also identified that 45 (46%) FBDG included beans in the animal group or protein source food group. Thirty-two (*n* = 32; 33%) included peanuts, and thirty-nine (40%) had soy in this group; 90% of the FBDGs incorporated eggs (*n* = 89; 90%) and fish (*n* = 89; 90%) into the group food from animals and only twenty FBDG (20%) included the seafood in that group. 

Furthermore, of the 235 terms used to identify the food items of each group, only 9% (*n* = 20) of the terms named foods from the milk and dairy product group: milk, cheeses, yogurts, dairy products, tofu, curds, labneh, foods rich in calcium, kefir (3%; *n* = 3) (Colombia, Estonia, and Hungary), soy milk (4%; *n* = 4) (USA, UK, Sweden, and El Salvador), tofu (2%; *n* = 2) (Cambodia and Yugoslavia), and eggs (3%; *n* = 3) (El Salvador, Guatemala, and Honduras). Only one (1%) FBDG (Oman) added dry curd (labneh) to this group. 

From a nutritional point of view, foods from animals are a source of proteins of high biological value as they provide all the essential amino acids. In addition, they are sources of complex B vitamins and lipids [83,96,97,100,101,102,103,104,105,106,107]. Proteins are macronutrients and a central part of the human diet, chemically consisting of carbon, hydrogen, nitrogen, and oxygen. Dietary proteins are found mainly in animal-derived food (milk and dairy products, meat, poultry, fish, and eggs), vegetables, and legumes. They occur in different proportions with varying amino acid profiles [83,97,102,103]. Proteins act in the constitution of any cell, are part of the composition of the body’s immune system antibodies, and actively participate in numerous metabolic processes and other body functions. Gluconeogenic amino acids are converted into glucose to provide energy [35,84,100,108,109,110]. 

Considering the lipid content, the literature reports the influence of the animal species, the type of handling, the diet, the cut type, the recipe, and the adopted preparation technique, among other factors [85,111]. 

#### 3.3.3. Fats and Sugar

Regarding the terms used to name the food items in the fat group, twenty-nine (29.6% of the 98 FBDG) terms were associated with oils and fats, e.g., butter, margarine, oils obtained from seeds, avocado, coconut, and olive oil. Fifteen FBDG (15.3%) included avocados in this group, while coconut was included in this group in ten (10.2%) of the FBDG (Appendix C). 

Twenty-two terms were used for food items in the sugar group. However, words like “highly processed foods rich in sugar and fat; oil; butter; fat; rice” are inconsistent with the proposed grouping (Appendix C). 

**Figure 5:**
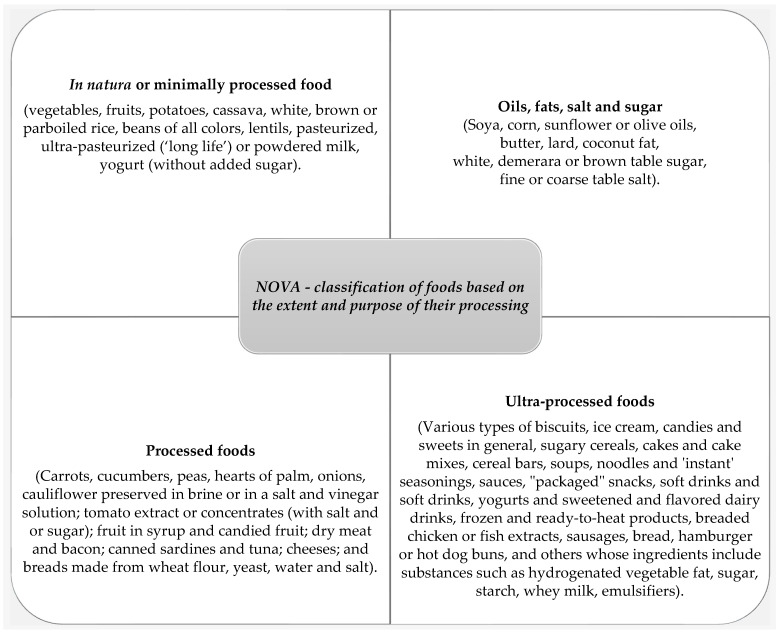
NOVA: A new classification of foods based on the extent and purpose of their processing.

In the FBDG from Brazil, Ecuador, and Uruguay, foods were classified according to the NOVA (classification of foods based on the extent and purpose of their processing), which ranks foods as in natura/minimally processed (Group 1); in Group 2—oils, fats, salt, and sugar (processed culinary ingredients), Group 3—processed foods, and Group 4—ultra-processed foods (Figure 5) [3,12,24,35,95,112,113,114,115]. This classification only makes it possible to compare epidemiological studies that used this same grouping, not reflecting the chemical composition and nutritional value of a given food item/product.

## 4. Points of Convergence between the Classification of Foods according to Food-Based Dietary Guidelines and the Food Science and Technology

**Figure 6:**
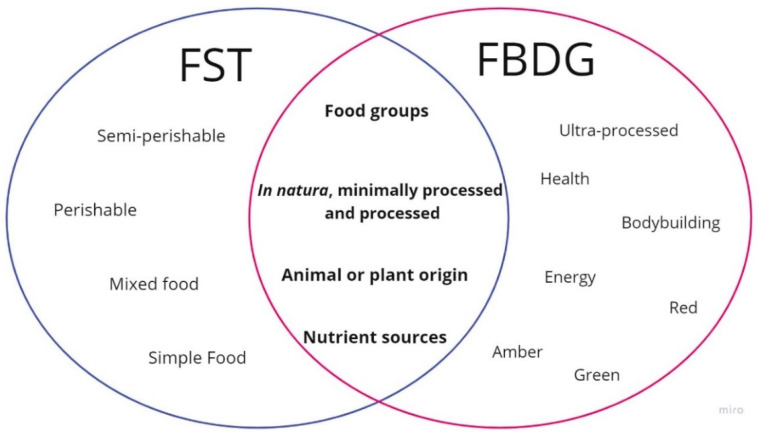
Possible points of the convergences between the classification of foods according to the Food-based Dietary Guidelines (FBDG) and food science and technology (FST).

The present study investigated convergence points between the classification of foods adopted by the researched Food-based Dietary Guidelines (FBDG) and the criteria adopted by food science and technology (FST). Our data show points of convergence when using criteria related to origin, nutrient sources, nature/processing, and food group (Figure 6). Our study identified inconsistencies in the classification recommended by the FDBG regarding FST due to the mistaken grouping of some food items in the groups, as sources of nutrients, as minimally processed foods, and in terms of origin, animal or vegetable.

The current trend is for FBDG to be based on the dietary pattern of the target group, not nutrients [43]. Based on studies of associations between the consumption of certain nutrients and the onset of diseases, approximately thirteen (*n* = 13; 13%) FBDG used terms associated with the classification according to nutrient sources.

The types of classification most adopted by the FBDG are those related to food groups and nutrient sources. However, most FBDG (94%) are classified according to food groups, a criterion also used by FST, rather than nutrient sources (Figure 6). A recent study with 2333 Brazilians from all Federative Units found that 54% (*n* = 1259) of these consumers believe it is easier to classify foods into food groups [12]. Even so, there is a variety of combinations between the food items included in these groups that must be rigorously analyzed. 

Regarding fruit and vegetable classification, approximately 49% of FBDG group fruits with vegetables. Fruits and vegetables, when correctly consumed in terms of frequency and quantity and combined with other foods, promote health benefits. Therefore, it is important to consider the analysis of the formulation, with the final nutritional composition of a product that uses fruits and vegetables as raw materials, as well as the addition of sugar, fat, and salt to these preparations [36,116,117]. In nutritional terms, fruits and vegetables differ in chemical composition and, consequently, in nutritional properties [116,117]. Aside from that, 22% of FBDG included fruit juice in the fruits and vegetable group. Most FBDG also have recommended restrictions concerning fruit juice intake, prioritizing the consumption of fresh fruit. Daily fruit juice consumption can increase the development of type 2 diabetes by up to 21% in predisposed individuals [118]. Fruit juice offers reduced fiber, increasing the speed of sugar absorption in the gastrointestinal tract and generating insulin spikes that can harm the body, for example [118,119,120]. 

Beans and other legumes are foods that are classified differently according to the different FBDG. They are included in the fruit and vegetable group, the cereals group, or even included in the food from animals group and with oilseeds. As we have already highlighted, these foods differ in chemical composition and nutritional properties. Legumes are known to be grains contained in pods; as they are grains, in 13% of the 98 FBDG, they are classified as cereals. However, legumes differ from cereals [77,83,97,117,121,122,123,124]. For this reason, 46% of FBDG probably included beans and/or pulses in the food from animals and dairy products group. It should be noted that the content and bioavailability of legume proteins differ from animal products (meat, milk, eggs, and derivatives) with proteins of higher biological value [83,97,103,110,124,125]. In the same way, some FBDGs have included soy milk or tofu in the animal food and dairy products group. They are plant-based products consumed by vegans, lactose intolerants, and those allergic to milk proteins, which should not be compared to foods of animal origin in terms of nutritional quality. Studies have shown that plant-based milk (rice, soy, and quinoa) may have the same protein content as bovine milk. However, the protein profile is significantly different, as well as the lower content and bioavailability of minerals (calcium) and anti-nutritional compounds, such as tannins. It does not validate the presence of this food in the group of foods of animal origin and dairy products [83,97,99,101,126]. It is noteworthy that it is not correct grouping legumes, a plant-based food, in the food from animal group based on protein content. 

Twenty-three (23.5%) FBDG adopted a specific legume group, justified by their nutritional properties and anti-nutritional factors. These modify their recommendation compared to other foods, such as cereals, meats, and oilseeds [117,122,124]. Except for methionine, legumes have all the amino acids, including lysine, which is a limiting amino acid for cereals. For this reason, it is recommended to combine the consumption of legumes and cereals in the same meal in an adequate proportion to increase their nutritional value [121]. 

Approximaely 40% of FBDG incorrectly included pulses, soy, and peanuts in the food from animals group. Both soy and peanuts are products of plant origin and do not have high biological value proteins. In addition, peanuts are legumes with a high fat content [103,123,124]. Approximately 11% of FBDG included oilseeds and seeds in the cereals and pulses group. Legumes have a lipid fraction predominance of monounsaturated and polyunsaturated fatty acids [121]. The oilseeds have a higher fat content consisting mainly of monounsaturated and polyunsaturated fatty acids, such as linoleic acid. They also have an essential content of vitamins B6 and E and minerals (selenium, magnesium, and potassium) and lower carbohydrates, proteins, and fiber content than legumes. Given this composition, it is not recommended to group oilseeds and seeds into the cereals and legumes group [104,121,123,124,127]. 

FBDGs of 17 Latin American countries added green bananas to the cereals and pulses group. In green bananas, 60–80% of the carbohydrates are fiber (resistant starch, celluloses, hemicelluloses, and lignin). Their ingestion is recommended since they behave in the body similarly to fibers and are considered healthy products [128,129]. 

Eggs, milk, and derivatives are products of animal origin with high nutritional quality recognized as important for human health. Their protein composition provides individuals the essential amino acids to develop and maintain vital activities [99,121,124,126]. Ninety-one (91%) FBDG grouped eggs into the food from animals group. However, six (6.2%) FBDG included sausage in the group of foods of animal origin since meat is the main ingredient in sausage. However, due to the large use of unhealthy additives and high amounts of sodium and fat, its consumption is associated with NCD risk [96,106,110]. Therefore, consuming meat products such as mortadella, sausage, salami, ham, and bacon is not encouraged and must be consumed cautiously [103,130,131]. 

The FBDG of Vietnam, Korea, Cambodia, and Kenya included in their dietary guidelines the consumption of insects in the protein-rich foods group, most likely due to the impact of the traditional intake of insects in these countries [103,130,131]. 

In the oils and fats group, almost 15 (15.3% (*n*= 15)) and 10 (10.2%) of the FBDG included avocado and coconut in this group, respectively. From the point of view of FST, avocado and coconut are fruits with high-fat content. It is known that oils (soybean, corn, rice, sesame, etc.), generally liquid at room temperature, have higher contents of monounsaturated and polyunsaturated fatty acids, except for coconut and palm oils. Olive oil is part of this group, but this term is only used to name oils from fruits: palm oil and olive oil. On the other hand, fats of animal origin, solid at room temperature, are the primary sources of saturated fatty acids such as bacon, lard, and dairy fat. Physiologically, they behave differently in the body; therefore, the fat type should be better specified in dietary recommendations [132,133,134]. 

Likewise, some contradictions were observed among 22 (9.3%) terms for the sugar group in the FBDG. Some authors consider “sugars” all sugars used as ingredients in processed and prepared foods such as bread, cakes, soft drinks, jams, chocolates, and ice cream. Total sugars are mono and disaccharides naturally present in food, such as lactose in milk, sucrose in table sugar, or glucose and fructose in honey. Table sugar is a product obtained from sugar cane or sugar beet. Such substances are used to impart sweetness to food, among other technological properties, including preserving food [61,85,135,136]. The term free sugar is used in industrialized foods that do not have sugar addition in their formulations. Added sugar refers to adding sugar to foods during processing or formulation preparation. Products with added sugar or sugars, such as sweeties, sweet snacks, and sweetened drinks, among others, have different levels of these substances in their formulations, making it difficult to safely assess how much of the product is a source of simple carbohydrates (mono- and disaccharides) [61,85,135,136].

Comparing the food groups described in the 98 FBDG, as well as the food items included in the groups, with the classification proposed by the FST, we identified that despite convergences regarding the sort, e.g., animal origin and vegetable origin, there is incompatibility regarding the inclusion of items in the groups (cereals, vegetables, vegetables; products from animal, insects, eggs, dairy products). FST follows the *Codex Alimentarius* recommendations, while the logic for classifying foods in the FBDG does not harmonize, for example, origin, group, and main source of nutrients, as verified for foods of plant origin and legumes. Despite identifying divergencies concerning food classification, it is essential to mention that FBDGs are designed for another purpose than a food composition table or the classification carried out by FST. However, guiding the population using correct and understandable classification is crucial to avoid misinterpretation.

The NOVA classification system groups foods as in natura or minimally processed, processed, and ultra-processed foods (Figure 3). Although NOVA classification is not considered FBDG or FST classification, NOVA classification is used to classify foods in the Brazilian FBDG. 

Considering the classification in terms of nature and food processing proposed by the FBDG and FST, we understand that they considered the same classification: in natura, minimally processed, and processed. However, the concepts proposed by FST differ from those established in the FBDG since minimally processed food, according to FST, is defined as any fresh fruit or vegetable or any combination that has been physically altered from its original form but remains fresh. The critical point of minimally processed fruits and vegetables is their active metabolism and respiratory rate despite physical changes [36,50,62,70,89,90]. Regardless of commodity, it has been trimmed, peeled, washed, and cut into 100% usable products. It is subsequently bagged or prepackaged to offer consumers high nutrition, convenience, and value while maintaining freshness. For the NOVA classification, minimally processed foods are in natura foods that have been subjected to cleaning processes, removal of inedible or undesirable parts, fractionation, milling, drying, fermentation, pasteurization, refrigeration, freezing, and similar techniques that do not involve adding salt, sugar, oils, fats, or other substances to the original food, diverging from the concepts described by FST [4,5,12,35,114,137].

Furthermore, in the FBDGs of Brazil, Peru, Ecuador, and Uruguay, the term ultra-processed is used to classify some foods. According to the NOVA classification, ultra-processed foods are industrial formulations made entirely or mainly from substances extracted from food (oils, fats, sugar, starch, and proteins), derived from food constituents (hydrogenated fats and modified starch), or synthesized in the laboratory based on organic materials such as petroleum and coal (colorants, flavorings, flavor enhancers, and various types of additives used to endow products with attractive sensory properties) [4,5,12,35,114,137]. It is essential to highlight that, in all instances, these terminologies (whether aligned with food standards or not) are aimed at supporting prudent food choices in population members, with some potential that they could be misconstrued due to the divergencies in the terminology. FST does not recognize this type of process/operation as a food classification. In recent years, the prevalence of NCD has increased, and many researchers have attributed this phenomenon to the consumption of industrialized foods [11,12,67]. Regarding processing as a means for classification, today, there is no consensus in the scientific community about the appropriateness of this approach. Some scientists view the NOVA classification as not contributing to new knowledge about food that has not been covered in the traditional way of classifying foods.

Industrialized foods can be added sugars, lipids, sodium, or food additives that can harm health when added improperly or when consumed in excess [12,70]. Similarly, processed foods in the household environment can be added to sugars, fats/oils, starches, proteins, natural pigments, seasonings and condiments, and others. Food processing is essential in providing edible, safe, and nutritious foods to the population and in food conservation. However, the topic is complex, with many processes that may bring risks and benefits depending on the context. There are reportedly negativity and misconceptions regarding processed foods in the media and by consumers [58,69,71,138].

Regarding the presentation of information on the classification of foods in the FBDG, some authors have shown that information on whether foods are sources of calcium, fat, or proteins still needs to be fully understood by the population [12,34,98,101,121,139]. The authors consider that consumers often need clarification on the information offered by different sources on nutrition and healthy lifestyles and even on the food composition (carbohydrates, fats, proteins, vitamins, and minerals) presented on labels [34]. In addition, there is a growing amount of incorrect information regarding food products published by the media, making it difficult to adopt healthy eating practices due to consumers’ difficulty differentiating between healthy and unhealthy [140,141,142]. The disparity of mistaken information is a factor that contributes to consumer disbelief regarding the safety and reliability of the product [12,30,143,144,145,146].

Advances in the labeling of industrialized products that identify, in addition to the product’s name, the list of ingredients, the nutritional information, and property claims on labels, provide better understanding, often restricted to professionals [11,12,36,145,147,148]. The dissemination of adequate messages about nutritional composition is essential to guide dietary practices [148]. In this context, messages must be short, clear, objective, easy to remember, understandable, and culturally acceptable to transform eating habits. According to some authors, FBDG should be practical and accessible, with many food options, to cater to different population groups and visual materials [12,34,40,41,42].

According to Sadler et al. [58], precise definitions (the appropriate description of a food item and its physical, chemical, and nutritional attributes and definition of what it is rather than what it is not) avoid multiple interpretations and enable a common understanding [58]. Healthy eating is associated with adequate intake, quantitatively and qualitatively, of nutrients. In addition to respecting cultural and social factors, it must consider combinations of food items and preparations and, consequently, the nutrients. Divergences regarding the indicator used as a reference for epidemiological studies can lead researchers to hasty conclusions and, possibly, inaccurate, given the mistakes considered in their selection [36].

Thus, to reduce information asymmetry between researchers, public policymakers, and consumers, using terms that nutritionally reflect food items for daily consumption is recommended, considering reliable data on their chemical composition that expresses the nutrition composition and sources of nutrients [36]. Public policymakers must know what are, at least, foods of animal or plant origins. Each *Codex Alimentarius* signatory country follows its recommendations, including the nomenclature of foods of animal and plant origin, whether in natura, minimally processed, or processed [2,149]. Humans have always used food as a function, predominantly of access to products and empirical knowledge, as well as the fact that the Industrial Revolution has provided significant behavioral changes in the world. Only part of the world population makes choices based on quality parameters, including nutritional quality, despite the demands of health, sustainability, authenticity, and ethics [12,33,38,58,65,69,150,151].

In the modern world, convenience is needed, and the food industry is essential. After approximately 200 years of the food industry’s existence and 60 years after food engineering became an established field of science, this has not been enough for some people to trust and feel safe with industrialized food. Furthermore, people lack knowledge about industrialized food, quality, and food safety, so how can they trust in something they do not know sufficiently? Including food subjects in basic education, such as food education, food safety, nourishment, good domestic food handling, and sustainability issues, must be considered in a public policy tool [138].

Despite the various proposals developed in epidemiological studies, Deharveng et al. [152] and Ireland et al. [9] support the need to define analytical methods to assess the chemical composition of food, standardize the “expression” of nutrients, and classify foods based on chemical composition data. In this direction, INFOODS (1997) clarifies the importance of having the chemical composition and, consequently, the nutritional value of in natura, minimally processed, or processed food at home or in the industry to group it in some group or category [153,154].

In addition to classifying foods, it is important to evaluate the chemical and nutritional composition of foods, simple and compound, since recipes, technical preparation files, and formulations are made up of a group of instructions related to the quantity and quality of raw materials and ingredients, to the precise recording of all ingredients, their proportions, and sequence of operations. Systematized recipes, technical preparation files, and formulations reveal foods’ chemical and nutritional composition and show trends in the relationship between food and nutrition [12,36]. The “formulations” originated from homemade recipes, initially comprised a list of ingredients and, later, began to describe the amount of each ingredient and the “how to do it.” With the upcoming food service, these recipes evolved into the technical preparation files, which, in this case, prioritize, in addition to economic aspects, the dish’s nutritional value [36,93]. Thus, in addition to understanding, it is necessary to discern the impact of the formulation, the food recipe on its chemical properties and its nutritional value, expressed in terms of energy or the content of a specific nutrient [36,155].

## 5. Conclusions

Our results showed that the classification of foods indicated in the 98 surveyed Food-based Dietary Guidelines (FBDG) established convergence with food science and technology (FST) for the criteria source of nutrients and origin of foods, animal or plant. By classifying foods in food groups in the FBDG, we identified that the distribution of some food items in food groups needs to follow the botanical classification (fruits, cereals, vegetables, and oleraceas) recommended by FST for foods of plant origin. A similar approach can be considered for foods of animal origin. Legumes do not have the same nutritional value as animal foods. Presumably, such items were incorporated into the group because they were protein-rich. Using such groupings can lead to erroneous interpretations regarding the chemical and nutritional aspects in the interpretation of food consumption versus nutritional quality. Regarding the nature of foods, whether in natura, minimally processed, or processed, from a scientific and technological point of view, it is impossible to classify foods according to the “level of processing”. Unit operations (cleaning, size reduction, mixing, adding ingredients, additives, heating, and packaging) do not define the nutritional value of the food.

A proposal for food classification should include eating habits, availability of products in the region, access issues (food security), technological development, and availability of data on chemical composition. We must consider and improve the consumers’ food literacy, informing them that foods, regardless of origin and technical process, are sources of nutrients. Indeed, its chemical composition determines whether a food item is a source of a particular nutrient. The chemical composition of a recipe or formulation will decide if the product is high in calories and is a source of lipids or fibers. The issue goes beyond a list that groups foods and is directly related to the correct information interpretation from the aspect of FST. The result of the recipe adopted at home, in food service, or in industry determines the processed food’s content, quality, and nutritional value. The results of this review reinforce an urgent need for shared work between professional nutritionists, food scientists and technologists, public policymakers, and representatives of the food industry to discuss the classification of foods. Just grouping foods into categories does not translate the nutritional value of a food or a product exactly. There is a need to further educate health workers and researchers regarding food classification; thus, they may not contribute to misinformation.

## Figures and Tables

**Table 2:** Continents and countries that have Food-based Dietary Guidelines (FBDG) according to FAO data [43].

Regions That Have Implemented the FBDG	Countries with Food-Based Dietary Guidelines	Total Countries	% Countries with FBDG
Africa	Benin, Ethiopia, Gabon, Kenya, Namibia, Nigeria, Seychelles, Sierra Leone, South Africa, and Zambia.	54	*n* = 10; 18%
Asia and the Pacific	Afghanistan, Australia, Bangladesh, Cambodia, China, Fiji, India, Indonesia, Japan, Malaysia, Mongolia, New Zealand, the Philippines, Republic of Korea, Sri Lanka, Thailand, and Vietnam.	50	*n* = 17; 34%
North America	Canada and the United States.	37	*n* = 2; 5%
Latin America and the Caribbean	Antigua and Barbuda, Argentina, Bahamas, Barbados, Belize, Bolivia, Brazil, Chile, Colombia, Costa Rica, Cuba, Dominica, Dominican Republic, Ecuador, El Salvador, Grenada, Guatemala, Guyana, Honduras, Jamaica, Mexico, Panama, Paraguay, Peru, Saint Kitts and Nevis, Saint Lucia, Saint Vincent, and the Grenadines, Uruguay, and Venezuela.	37	*n* = 29; 78%
Europe	Albania, Austria, Belgium, Bosnia and Herzegovina, Bulgaria, Croatia, Cyprus, Denmark, Estonia, Finland, France, Georgia, Germany, Greece, Hungary, Iceland, Ireland, Israel, Italy, Latvia, Malta, the Netherlands, Poland, Portugal, Romania, Republic of Moldova, Slovenia, Spain, Switzerland, Sweden, The former Yugoslav Republic of Macedonia, Turkey, and the United Kingdom.	50	*n* = 34; 68%
Near East	Iran, Lebanon, Oman, Qatar, Saudi Arabia, and the United Arab Emirates	14	*n* = 6; 43%

## Appendix A

**Table A1:** Selection of studies on food classification systems from 1997 to 2015.

Year	Authors	Objectives	Food Classification System
1997	Navas, Rebecchi, and Trombini [156]	To identify the dietary patterns prevalent in Europe and their sociodemographic determinants.	The DAFNE food classification system grouped food into cereals and cereal products; meat and meat products; red meat; offals; canned meat and meat products; meat dishes; fish and seafood; milk and milk products; eggs; lipids of animal origin; lipids of vegetable origin; potatoes and other starchy roots; pulses; vegetables; fresh vegetables; processed vegetables; nuts; fruits; fresh fruits and processed fruits; sugar and sugar products; non-alcoholic beverages; stimulants; mineral water; soft drinks; alcoholic beverages.
1999	Deharveng et al. [152]	To compare availability and define analytical methods and mode of expression of nutrients of interest for EPIC.	European Prospective Investigation into Cancer and Nutrition (EPIC)—Food composition tables from the following countries: Denmark, France, Germany, Greece, England, Italy, Holland, Spain, and Sweden.
2001	Lagiou et al. [27]	To assess European dietary patterns using household budget surveys (HBSs) data.	The DAFNE food classification system.
2002	Brussaard et al. [157]	To discuss the general outcome and conclusions of a European project (EFCOSUM); to develop a European food consumption survey method that delivers internationally comparable data on a set of policy-relevant nutritional indicators.	Discussion groups based on prior experiences such as DAFNE, EPIC, FLAIR Eurofoods Enfant project, COST Action 99, etc.
2002	Ireland et al. [9]	To harmonize food classification and food composition databases, allowing comparability of consumption at both food and nutrient levels in Europe.	EFG: 4 food groups (bread, vegetables (excluding potatoes), fruits (excluding fruit juice), and fish and seafood).
2011	EFSA [14]	To develop a standardized food classification and description system with general applicability and a preliminary technical system specification	FoodEx-2.
2011	Monteiro et al. [46]	To develop a classification of food purchases made by Brazilian households and explore the potential impact on the overall diet quality.	The group created a new classification of food based on the extent and purpose of food processing. The groups are unprocessed/minimally processed foods (Group 1), processed culinary ingredients (Group 2), and ultra-processed ready-to-eat or ready-to-heat food products (Group 3).
2012	Eicher-Miller et al. [47]	To develop food categories by level of processing to determine the contribution of processed food to the total daily intake of dietary nutrients.	Food categories based on various USDA composition tables. The categories are: minimally processed foods processed for preservation, mixtures of combined ingredients, ready-to-eat processed foods, or prepared foods/meals. The “mixtures of combined ingredients” category was separated into two subcategories: “packaged mixes and jarred sauces” and “mixtures probably home prepared.” The ready-to-eat processed foods” category was divided into two subcategories: “packaged ready-to-eat foods” and “mixtures possibly stored”.
2014	Weaver et al. [92]	To analyze the contribution of processed food to the US diet and review emerging technologies and the research needed to understand better the role of processed foods in a healthy diet.	The group used categories of processed foods as proposed by the International Food Information Council. The categories are: foods that require processing or production (also called “minimally processed”); foods processed to help preserve and enhance nutrients and freshness of foods at their peak; foods that combine ingredients such as sweeteners, spices, oils, flavors, colors, and preservatives with improving safety and taste and add visual appeal; “ready-to-eat” foods needing minimal or no preparation; foods packaged to stay fresh and save time.
2014	Moubarac et al. [25]	To evaluate food classification systems using a systematic review as follows: in Europe, the International Agency for Research on Cancer (IARC) used a methodology devised for the European Prospective Investigation into Cancer and Nutrition (EPIC) study;in the United States, the International Food Information Council Foundation (IFIC); in Mexico, the National Institute of Public Health in Mexico distinguishes between industrialized and local food and products and between modern and traditional foods and products.	Three main groups are identified: “non-processed foods”, consumed raw without any further processing; “modestly/moderately processed foods”, sub-divided into industrial and commercial foods that are finished with no additional cooking and foods processed at home and prepared/cooked from raw foods or moderately processed foods; “processed foods, sub-divided into “staple/basic foods” and “highly-processed foods.” Foods and products are classified as “minimally processed,”; “foods processed for preservation,”; “mixtures of combined ingredients,”; “ready-to-eat processed foods,”; “prepared foods/meals.” Three categories are used to describe food: “industrialized modern foods”, “industrialized traditional foods”, and “non-industrialized foods”, sub-divided into “modern and traditional preparations made from home”, “traditional preparations made at home or by artisanal”, and “unprocessed foods.”
2015	Poti et al. [113]	To determine 2000–2012 trends in the US households’ contribution to processed and convenience food categories, comparing saturated fat, sugar, and sodium content.	Four categories were created based on the degree of industrial food processing: unprocessed and minimally processed; basic processed; moderately processed; highly processed. Three types were also demonstrated based on product convenience.
2015	PAHO/WHO [158]	To show trends in ultra-processed food and drink product sales in 13 Latin American countries (Argentina, Bolivia, Brazil, Chile, Colombia, Costa Rica, Dominican Republic, Ecuador, Guatemala, Mexico, Peru, Uruguay, and Venezuela).	PAHO recommends the NOVA system: (1) unprocessed or minimally processed foods; (2) processed culinary ingredients; (3) processed foods; (4) ultra-processed food and drink products.

## Appendix B

**Table A2:** Terms used to name food items from the groups of vegetables, fruits, cereals, and legumes in the researched FBDG (*n* = 98).

Terms Used to Describe Food Items in the Fruit Group	Terms Used to Describe Food Items in the Vegetable Group	Terms Used to Describe Food Items from the Cereals Group	Terms Used to Describe Food Items from the Legume Group
Fruits and Vegetables	Fruits and Vegetables	Cereals and Tubers; Starchy Foods; Cereals and Cereal Products; Bread, Grains and Tubers; Rice, Bread, Cereals, Pasta, and Tubers; Grains, Roots, Tubers; Grain (Cereal) Foods; Rice, Bread, and Other Cereals; Cereals and Starchy Foods; Cereals, Tubers, and Legumes; Energy; Cereals, Millets and Pulses; Staple Foods/Staples; Grain; Rice, Noodle, Bread, Cereals, Cereals Products, and Tubers; Bread and Cereals; Rice, Rice Products, Corn, Root Crops, Bread, and Noodles; Rice, Breads, Other Cereals, and Yams; Rice, Rice Products, Other Grains, and Starchy Foods; Breads and Cereals; Cereals; Cereals, Grains, and Potatos; Cereals and Starchy Vegetables; Grain Products; Wholegrain Cereal Products; Cereals, Cereal Products, Potatoes, and Rice; Cereals and Granular Plants; Potato, Bread, Rice, Pasta, and Other Starchy Carbohydrates; Grains, Potatoes, and Pulses; Wholegrain; Wholegrain Cereals and Products; Cereals, Cereal Products, and Others Carbohydrate Foods; Cereals and Derivatives, Tubers; Bread, Grain Products, and Potatoes; Wholegrain Products and Potatoes; Wholemeal Cereals and Breads, Potatoes, Pasta, and Rice; Cereals, Cereal Products, and Potatoes; Cereals and Potatoes; Legumes, Cereals, Papa, Bread, and Pastas; Cereals, Legumes, Tubes, and Derivatives; Cereals, Roots, Tubes, Bananas, and Derivatives; Cereals, Legumes, and Starchy Vegetables; Cereals and Root Vegetables; Cereals, Grains, and Roots; Cereals, Grains, and Tubers; Starchy, Grains, and Cereals; Cereals, Tubers, and Derivatives; Cereals, Grains, Tubers, and Banana/Plantain; Cereals Grains, Roots, and Tubers; Bread, grain/cereal products, and potatoes; Wholegrain and Legumes; Cereals, Starchy Roots, and Tubers.	Legumes, Pulses, Nuts, and Seeds; Beans, Peas and Lentilis; Dry Beans, Peas, Lentils and Soya; Vegetables and Legumes; Pulses; Legumes; Peas, Beans, and Nuts; Beans and Peas; Legumes/Nuts; Meat and Legumes; Legumes, Pulses, and Nuts.
Vegetables, Berries, and Fruits	Vegetables, Berries, and Fruits		
Vegetables, Salad, and Fruit	Vegetables, Salad, and Fruit		
Vegetables, Legumes, and fruits	Vegetables, Legumes, and Fruits		
Fruits, Herbs, and Vegetables	Fruits, Herbs, and Vegetables		
Fruits, Green Leaves and Vegetables	Fruits, Green Leaves and Vegetables		
Health	Health		
Fruits	Vegetables		
	Vegetables and Sauces Based on Vegetables		
	Vegetables and Dark Green Leafy Vegetables		
	Vegetables and Legumes		
	Vegetables and Salad		
	Vegetables and Tubers		

## Appendix C

**Table A3:** Terms used to name food items from the groups of meat, dairy products, eggs, fats, and sugars in the researched FBDG (*n* = 98).

Terms Used to Name Food Items in the Meat Group	Terms Used to Name Food Items in the Dairy Products Group	Terms Used to Name Food Items in the Fat Group	Terms Used to Name Food Items in the Sugars Group
Meat, Fish, Beans, and Other Sources of Protein; Meat, Fish, and Animal Protein Products; Animal Source Foods and Beans; Eggs, Fish, Meat, and Dairy; Fish, Meat, and Alternatives; Fish, Poutry, Meat, Milk, and Eggs; Chicken, Fish, Meat, and Eggs; Meat, Fish, and Eggs; Lean Meats and Poultry, Fish, Eggs, Tofu and Seeds, and Legumes/Beans; Meat, Fish, Eggs, and Beans; Lean Meats; Body Building; Milk and Animal Foods; High-Protein Foods; Fish, Poutry, Meat and Legumes, Fish and Meat Dishes; Lean Meats, Chicken, Seafood, Eggs, Legumes, Nuts, and Seeds; Fish, Shelfish, Meat, and Poultry, Dried Beans, and Nuts; Fish, Pulses, Meat, and Eggs; Milk, Fish, Lean Meats, Eggs, Legumes, and Pulses; Protein-Rich Foods; Meats and Eggs; Lean Meats, Eggs, Legumes, and Unsalted Nuts and Seeds; Fish, Poultry, Meats, Eggs, and Alternatives; Meat and Alternatives; Protein; Meat, Poultry, Fish, Eggs, and Dried Legumes; Beans, Pulses, Fish, Eggs, Meat, and Other Proteins; Meat, Eggs, Fish, Legumes, and Seeds; Dairy Products, Meat, Fish, Eggs, and Tofu; Red and Processed Meat; Fish and Shellfish; Fish, Poultry, Pulses, Nuts, Eggs, Red Meat, and Meat Products; Meat and Fish; Meat Preparations; Meat, Fish, Seafood, and Eggs; Fish, Legumes, Meat, Egg, Nuts, and Dairy; Lean Meat, Fish, Poultry, Eggs, Legumes, Nuts, and Seeds; Lean Meat, Fish, Eggs, Pulses, Nuts, and Seeds; Meat, Poultry, Fish, Eggs, Beans, and Nuts; Meat, Red Meat, White Meat, and Poultry, Eggs, Fish, and Seafood; Meat, Sausage, Fish, and Eggs; Meat, Poultry, Fish, Fish Products, and Eggs; Fish, Poultry, Eggs, Meat, and Meat Products; Meat and Meat Toppings; Animal Source Foods; Meat, Fish, Eggs, Pulses, and Nuts; Meat, Fish, Eggs, and Meat Alternatives; Meat, Meat By-Products, Fish, Eggs, Kidney, Beans, Nuts, etc.; Food From Animals; Meats and Dairy; Meat, Derivatives, Eggs, and Vegetable Mixtures; Meat, Legumes, and Eggs; Meat, Eggs, Pulses, Nuts, and Seeds; Meat, Fish, Poultry, Eggs, and Beans; Meat, Fish, Poultry, Eggs, Organ Meat, Milk, and Dairy Products; Poultry, Fish, Beef, Organ Meat, or Menudos; Food From Animals and Legumes; Dairy, Meat, and Eggs; Meat Products, Fish, Eggs, and Legumes; Meat and Legumes; Fish, Insects and Animal-Source Foods.	Dairy Products; Milk and Milk Products; Milk, Maas, and Yohurt; Dairy; Milk, Yoghurt, Cheese, and/or Their Alternatives; Milk and Dairy Products; Body Building; Milk; Low-Fat Milk and Dairy Products; Milk and Dairy; Milk, Dairy Products, and Alternatives; Milk and Alternatives; Milk Products; Dairy and Alternatives; Low-Fat Dairy Products; Milk, Yogurt, and Cheese; Dary and Calcium-Enriched Products; Milk and its By-Products; Eggs, Milk, and Dairy Products.	Fats and oils, sweeties and butter; Olive Oil; Highly processed foods rich in sugar and fat; Oil, butter, sweets, and confectionery; Other animal sources; products and nuts; High-fat foods; Fats, spreads, and oils; Softer and healthier fat; Nuts, Seeds, Oil Vegetables; Oils and fatty products; Processed Foods High in Fat, Sugar, and Salt; Nuts and Oil Seed; Fats, Oils, and Sugar; Foods Containing Fats; Foods Containing Sugar; Fats and Oils; Oils, Dried Fruits, and Seeds/*Aceites; Frutas Secas y Semillas*; Optionals: Sweets and Fats/*Opcionales: Dulces y Grasas*; Oils, Fats, Salt, and Sugar; *Grasas y otros alimentos fuentes de grasas*/Fats or Other Sources of Fat; *Grasas, azúcar y sal yodada*/Fats, Sugar, and Iodized Salt; *Aceites y azucares*/Oils and Sugars; *Aceites, grasas y azucares*/Oils, Fats, and Sugars; Fats and Sugars; Oils; Energy; Amber; Fats; Spreading and Cooking Fats.	Optional: Sweets and Fats/*Opcionales: Dulces y Grasas; Azúcares y Mermeladas*/Sugars and Marmalades; Oils, Fats, Salt, and Sugar; *Azúcar*/Sugar/Sweets; *Azucares y Mieles*/Sugars and Honeys; Sugars and Sweets; Sweet, Fatty, and Salty Products; Fats and Sugars, Fats and Oils, and Sweeties and Butter; Sweet/Salty Snacks; Sweets, Snacks, and Sweetened Beverages; Highly Processed Foods Rich in Sugar and Fat; Oil, Butter, Sweets, and Confectionery; Sweets, Snacks, and Sweetened Drinks; Products Sucres/Sweet Products; Sugar and Confectionery and Pastry; Sugary Products; Processed Foods High In Fat, Sugar, and Salt; Rice, Cereals, Starchy Foods, and Sugar; Sugar and Sweets; Fats, Oils, and Sugar; Foods Containing Fats; Foods Containing Sugar.

## References

[1] Balakrishna Y., Manda S., Mwambi H., van Graan A. (2021). Identifying Nutrient Patterns in South African Foods to Support National Nutrition Guidelines and Policies. Nutrients.

[2] FAO (1998). WHO Preparation and Use of Food-Based Dietary Guidelines.

[3] Marino M., Puppo F., Del Bo’ C., Vinelli V., Riso P., Porrini M., Martini D. (2021). A Systematic Review of Worldwide Consumption of Ultra-Processed Foods: Findings and Criticisms. Nutrients.

[4] Monteiro C.A., Cannon G., Levy R.B., Moubarac J.C., Louzada M.L.C., Rauber F., Khandpur N., Cediel G., Neri D., Martinez-Steele E. (2019). Ultra-Processed Foods: What They Are and How to Identify Them. Public Health Nutr..

[5] Steele E.M., O’Connor L.E., Juul F., Khandpur N., Galastri Baraldi L., Monteiro C.A., Parekh N., Herrick K.A. (2022). Identifying and Estimating Ultraprocessed Food Intake in the US NHANES According to the Nova Classification System of Food Processing. J. Nutr..

[6] Yang Y.X., Wang X.L., Leong P.M., Zhang H.M., Yang X.G., Kong L.Z., Zhai F.Y., Cheng Y.Y., Guo J.S., Su Y.X. (2018). New Chinese Dietary Guidelines: Healthy Eating Patterns and Food-Based Dietary Recommendations. Asia Pac. J. Clin. Nutr..

[7] Wu W., Zhang A., van Klinken R.D., Schrobback P., Muller J.M. (2021). Consumer Trust in Food and the Food System: A Critical Review. Foods.

[8] Ireland J.D., Møller A. (2000). Review of International Food Classification and Description. J. Food Compos. Anal..

[9] Ireland J.D., van Erp-Baart A.M.J., Charrondiere U.R., Moller A., Smithers G., Thichopoulou A., Gruop E. (2002). Selection of a Food Classification System and a Food Composition Database for Future Food Consumption Surveys. Eur. J. Clin. Nutr..

[10] Ireland J.D., Møller A., Caballero B., Finglas P.M., Toldrá F.B.T.-E. (2005). Food Classification and Description.

[11] Petrus R.R., do Amaral Sobral P.J., Tadini C.C., Gonçalves C.B. (2021). The NOVA Classification System: A Critical Perspective in Food Science. Trends Food Sci. Technol..

[12] Monteiro J.S., Nakano E.Y., Zandonadi R.P., Botelho R.B.A., Araujo W.M.C. (2022). How Do Consumers Understand Food Processing? A Study on the Brazilian Population. Foods.

[13] Møller A., Kearney J., De Henauw S., Steingrı L., Brussaard J.H., Lo M.R.H. (2002). A European Food Consumption Survey Method—Conclusions and Recommendations. Eur. J. Clin. Nutr..

[14] EFSA (2011). Report on the Development of a Food Classification and Description System for Exposure Assessment and Guidance on Its Implementation and Use 1. EFSA J..

[15] Ramsingh B. (2012). The History of International Food Safety Standards and the Codex Alimentarius (1955–1995). Ph.D. Thesis.

[16] Foundation L., Series W.P., Seaton M.B., Allin S. (2007). Mcis Briefings—Comparative Program on Health and Society Lupina Foundation Working Papers Series 2007–2009.

[17] FAO, WHO (1994). Joint FAO/WHO Program on Food Standards.

[18] FAO, WHO CODEX Alimentarius—International Foods Standards. https://www.fao.org/fao-who-codexalimentarius/about-codex/history/en/.

[19] Kalansooriya C., Jofre-Bonet M., Serra-Sastre V. (2023). Nutritional Transition, Demand for a Healthy Diet and the Role of Education: An Analysis Using Almost Ideal Demand System. Demand for a Healthy Diet and the Role of Education: An Analysis Using Almost Ideal Demand System.

[20] Elechi J.O.G., Sirianni R., Conforti F.L., Cione E., Pellegrino M. (2023). Food System Transformation and Gut Microbiota Transition: Evidence on Advancing Obesity, Cardiovascular Diseases, and Cancers—A Narrative Review. Foods.

[21] Steyn N.P., Mchiza Z.J. (2014). Obesity and the Nutrition Transition in Sub-Saharan Africa. Ann. N. Y. Acad. Sci..

[22] Jiang K., Zhang Z., Fullington L.A., Huang T.T., Kaliszewski C., Wei J., Zhao L., Huang S., Ellithorpe A., Wu S. (2022). Dietary Patterns and Obesity in Chinese Adults: A Systematic Review and Meta-Analysis. Nutrients.

[23] De Fragas Hinnig P., Monteiro J.S., de Assis M.A.A., Levy R.B., Peres M.A., Perazi F.M., Porporatti A.L., Canto G.D.L. (2018). Dietary Patterns of Children and Adolescents from High, Medium and Low Human Development Countries and Associated Socioeconomic Factors: A Systematic Review. Nutrients.

[24] Sarmiento-Santos J., Souza M.B.N., Araujo L.S., Pion J.M.V., Carvalho R.A., Vanin F.M. (2022). Consumers’ Understanding of Ultra-Processed Foods. Foods.

[25] Moubarac J.-C., Parra D.C., Cannon G., Monteiro C.A. (2014). Food Classification Systems Based on Food Processing: Significance and Implications for Policies and Actions: A Systematic Literature Review and Assessment. Curr. Obes. Rep..

[26] Marconi S., Durazzo A., Camilli E., Lisciani S., Gabrielli P., Aguzzi A., Gambelli L., Lucarini M., Marletta L. (2018). Food Composition Databases: Considerations about Complex Food Matrices. Foods.

[27] Lagiou P., Trichopoulou A., Henderickx H.K., Remaut-de Winter A.M., Buermans H., Merckx L., Kaíc-Rak A., Antonic-Degac K., Hrabak-Zerjavic V., Grubisic M. (2001). The DAFNE Initiative: The Methodology for Assessing Dietary Patterns across Europe Using Household Budget Survey Data. Public Health Nutr..

[28] Schlotke F., Becker W., Ireland J.D., Anders M.A., Ovaskainen B., Monspart J., Unwin I. (2000). EUROFOODS Recommendations for Food Composition Database Management and Data Interchange. J. Food Compos. Anal..

[29] Charrondiere U.R., Burlingame B. (2007). Identifying Food Components: INFOODS Tagnames and Other Component Identification Systems. J. Food Compos. Anal..

[30] Aydinoğlu N.Z., Krishna A. (2011). Guiltless Gluttony: The Asymmetric Effect of Size Labels on Size. J. Consum. Res..

[31] Dornyei K.R., Gyulavari T. (2016). Why Do Not You Read the Label ?—An Integrated Framework of Consumer Label Information Search. Int. J. Consum. Stud..

[32] Marano-Marcolini C., Torres-Ruiz F.J. (2017). A Consumer-Oriented Model for Analysing the Suitability of Food Classification Systems. Food Policy.

[33] Torres-Ruiz F.J., Marano-Marcolini C., Lopez-Zafra E. (2018). In Search of a Consumer-Focused Food Classi Fi Cation System: An Experimental Heuristic Approach to Di Ff Erentiate Degrees of Quality. Food Res. Int..

[34] Montagnese C., Santarpia L., Buonifacio M., Nardelli A., Caldara A.R., Silvestri E., Contaldo F., Pasanisi F. (2015). European Food-Based Dietary Guidelines: A Comparison and Update. Nutrition.

[35] Ministério da Saúde (2014). Dietary Guidelines for the Brazilian Population.

[36] Botelho R., Araújo W., Pineli L. (2018). Food Formulation and Not Processing Level: Conceptual Divergences between Public Health and Food Science and Technology Sectors. Crit. Rev. Food Sci. Nutr..

[37] Bisogni C.A., Jastran M., Seligson M., Thompson A. (2012). Special Article How People Interpret Healthy Eating: Contributions of Qualitative Research. J. Nutr. Educ. Behav..

[38] Blake C.E.Ã., Bisogni C.A., Sobal J., Devine C.M., Jastran M. (2007). Classifying Foods in Contexts: How Adults Categorize Foods for Different Eating Settings. Appetite.

[39] Rodriguez-Oliveros M.G., Bisogni C.A., Frongillo E.A. (2014). Knowledge about Food Classification Systems and Value Attributes Provides Insight for Understanding Complementary Food Choices in Mexican Working Mothers. Appetite.

[40] Montagnese C., Santarpia L., Iavarone F., Strangio F., Caldara A.R., Silvestri E., Contaldo F., Pasanisi F. (2017). North and South American Countries Food-Based Dietary Guidelines: A Comparison. Nutrition.

[41] Bechthold A., Boeing H., Tetens I., Schwingshackl L., Nöthlings U. (2018). Perspective: Food-Based Dietary Guidelines in Europe—Scientific Concepts, Current Status, and Perspectives. Adv. Nutr..

[42] Albert J.L., Samuda P.M., Molina V., Regis T.M., Dietetics D., Severin M., Finlay B., Prevost J.L. (2007). Developing Food-Based Dietary Guidelines to Promote Healthy Diets and Lifestyles in the Eastern Caribbean. J. Nutr. Educ. Behav..

[43] FAO Food-Based Dietary Guidelines. http://www.fao.org/nutrition/education/food-dietary-guidelines/background/en/.

[44] Dos Santos Chagas C.M., Botelho R.B.A., Toral N. (2018). Healthy Eating through the Eyes of Adolescents: A Qualitative Analysis of Messages from the Dietary Guidelines for the Brazilian Population. Rev. Nutr..

[45] Truswell A.S., Bateson D.J., Madafiglio K.C., Pennington J.A.T., Rand W.M., Klensin J. (1991). INFOODS Guidelines for Describing Foods: A Systematic Approach to Describing Foods to Facilitate International Exchange of Food Composition Data. J. Food Compos. Anal..

[46] Monteiro C.A., Levy R.B., Claro R.M., De Castro I.R.R., Cannon G. (2011). Increasing Consumption of Ultra-Processed Foods and Likely Impact on Human Health: Evidence from Brazil. Public Health Nutr..

[47] Eicher-Miller H.A., Fulgoni V.L., Keast D.R. (2012). Contributions of Processed Foods to Dietary Intake in the US from 2003–2008: A Report of the Food and Nutrition Science Solutions Joint Task Force of the Academy of Nutrition and Dietetics, American Society for Nutrition, Institute of Food Technologi. J. Nutr..

[48] Albala K. (2013). Food: A Cultural Culinary History.

[49] Giuntini E.B., Lajolo F.M., Menezes E.W. (2006). De Composição de Alimentos: Um Pouco de História. Arch. Latinoam. Nutr..

[50] Knorr D., Watzke H. (2019). Food Processing at a Crossroad. Front. Nutr..

[51] Koivistoinen P.E. (1996). Introduction: The Early History of Food Composition Analysis-Source of Artifacts until Now. Food Chem..

[52] Mcmasters V. (1963). History of Food Composition Tables of the World. J. Am. Diet Assoc..

[53] Welch R.W., Mitchell P.C. (2000). Food Processing: A Century of Change. Br. Med. Bull..

[54] Carpenter K.J. (2003). A Short History of Nutritional Science: Part 1 (1785–1885). J. Nutr..

[55] Carpenter K.J. (2003). A Short History of Nutritional Science: Part 2 (1885–1912). J. Nutr..

[56] Carpenter K.J. (2003). A Short History of Nutritional Science: Part 3 (1912–1944). J. Nutr..

[57] Carpenter K.J. (2003). A Short History of Nutritional Science: Part 4 (1945–1985). J. Nutr..

[58] Sadler C.R., Grassby T., Hart K., Raats M., Sokolović M., Timotijevic L. (2021). Processed Food Classification: Conceptualisation and Challenges. Trends Food Sci. Technol..

[59] FAO, WHO (1993). Classification of Foods and Feeds.

[60] Amit S.K., Uddin M.M., Rahman R., Islam S.M.R., Khan M.S. (2017). A Review on Mechanisms and Commercial Aspects of Food Preservation and Processing. Agric. Food Secur..

[61] Fellows P.J. (2009). Food Processing Technology: Principles and Practice.

[62] Floros J.D., Newsome R., Fisher W., Barbosa-Cánovas G.V., Chen H., Dunne C.P., German J.B., Hall R.L., Heldman D.R., Karwe M.V. (2007). Feeding the World Today and Tomorrow: The Importance of Food Science and Technology An IFT Scientific Review. Compr. Rev. Food Sci. Food Saf..

[63] Knorr D., Augustin M.A. (2021). Food Processing Needs, Advantages and Misconceptions. Trends Food Sci. Technol..

[64] Priyadarshini A., Rajauria G., O’Donnell C.P., Tiwari B.K. (2019). Emerging Food Processing Technologies and Factors Impacting Their Industrial Adoption. Crit. Rev. Food Sci. Nutr..

[65] Sammugam L., Pasupuleti V.R. (2019). Balanced Diets in Food Systems: Emerging Trends and Challenges for Human Health. Crit. Rev. Food Sci. Nutr..

[66] Van Boekel M., Fogliano V., Pellegrini N., Stanton C., Scholz G., Lalljie S., Somoza V., Knorr D., Jasti P.R., Eisenbrand G. (2010). A Review on the Beneficial Aspects of Food Processing. Mol. Nutr. Food Res..

[67] Carretero C., Clotet R., Colomer Y., De G.G. (2020). Food Classification Report: The Concept “Ultra—Processed”.

[68] Lawrence A., Jones H.J. (2017). Exploring Australian’s Attitudes towards Five Food Group Foods and Discretionary Choices. J. Nutr. Intermed. Metab..

[69] Meijer G.W., Lähteenmäki L., Stadler R.H., Weiss J. (2021). Issues Surrounding Consumer Trust and Acceptance of Existing and Emerging Food Processing Technologies. Crit. Rev. Food Sci. Nutr..

[70] Rego R.A., Vialta A., Madi L. (2018). Industrialized Foods: The Importance for Brazilian Society.

[71] Kumar V., Chandra S., Kumar K., Goyal S.K., Kumar L., Kumar A. (2017). Perishable and Non-Perishable Food Products Roles in Environment—A Review. South Asian J. Food Technol. Environ..

[72] Domingos A. (2006). Handbook of Horticultural Crops–Volume I.

[73] Domingos A. (2006). Handbook of Horticultural Crops–Volume II.

[74] Bevilacqua H.E.C.R. (2008). Classificação das Hortaliças. Horta: Cultivo de Hortaliças.

[75] Filgueira F.A.R. (2000). New Horticultural Manual.

[76] Gopalakrishnan T.R. (2007). Vegetable Crops.

[77] Vaughan J., Geissler C. (2009). The New Oxford Book of Food Plants.

[78] Brazil Resolution-RDC No. 43, of 1 September 2015. https://bvsms.saude.gov.br/bvs/saudelegis/anvisa/2015/rdc0043_01_09_2015.pdf.

[79] Brennan J.G., Grandison A.S. (2011). Food Processing Handbook. Vol. I.

[80] de Guiné R.P.F., dos Corrreia P.M.R. (2014). Engineering Aspects of Cereal and Cereal-Based Products.

[81] FAO, WHO (1989). Codex Standard for Certain Pulses.

[82] Quintieri L., Nitride C., De Angelis E., Lamonaca A., Pilolli R., Russo F., Monaci L. (2023). Alternative Protein Sources and Novel Foods: Benefits, Food Applications and Safety Issues. Nutrients.

[83] Romão B., Botelho R.B.A., Torres M.L., da Costa Maynard D., Machado de Holanda M.E., Borges V.R.P., Raposo A., Zandonadi R.P. (2023). Nutritional Profile of Commercialized Plant-Based Meat: An Integrative Review with a Systematic Approach. Foods.

[84] FAO, WHO, FAO–Food and Nutrition 51 (1990). Protein Quality Evaluation.

[85] Araujo W.M.C., Montebello N.D.P., Botelho R.B.A., Borgo L.A. (2014). Alquimia dos Alimentos. Teoria e Prática.

[86] Brazil Resolution No. 005, of 5 July 1991. https://sidago.agrodefesa.go.gov.br/site/adicionaisproprios/protocolo/arquivos/409894.pdf.

[87] United Nations Economic Commission for Europe (1986). UNECE Standards for Eggs and Egg Products.

[88] FAO, WHO General Standard for the Use of Dairy Terms. https://www.fao.org/fao-who-codexalimentarius/sh-proxy/en/?lnk=1&url=https%253A%252F%252Fworkspace.fao.org%252Fsites%252Fcodex%252FStandards%252FCXS%2B206-1999%252FCXS_206e.pdf.

[89] De Oliveira Silva E., Pinto P.M., Jacomino A.P., Torres Silva L. Processamento Mínimo de Produtos Hortifrutícolas. https://ainfo.cnptia.embrapa.br/digital/bitstream/item/54160/1/DOC11007.pdf.

[90] Kopf C. (2008). Técnicas Do Processamento de Frutas Para a Gricultura Familiar.

[91] Charrondiere R., Stadlmayr B., Rittenschober D., Grande F., Nowak V. (2017). FAO/INFOODS Food Composition Database for Biodiversity Version 4.0-–BioFoodComp4.0.

[92] Weaver C.M., Dwyer J., Iii V.L.F., King J.C., Leveille G.A., Macdonald R.S., Ordovas J., Schnakenberg D. (2014). Processed Foods: Contributions to Nutrition 1,2. Am. J. Clin. Nutr..

[93] De Cássia Akutsu R., Botelho R.A., Camargo E.B., Sávio K.E.O., Araújo W.C. (2005). A Ficha Técnica de Preparação Como Instrumento de Qualidade Na Produção de Refeições. Rev. Nutr..

[94] Charrondiere U., Rittenschober D., Nowak V., Nicodemi C., Bruggeling P., Petracchi C. (2016). FAO/INFOODS e-Learning Course on Food Composition Data. Food Chem..

[95] Dos Santos Costa C., de Faria F.R., Gabe K.T., Sattamini I.F., Khandpur N., Leite F.H.M., Steele E.M., da Costa Louzada M.L., Levy R.B., Monteiro C.A. (2021). Escore Nova de Consumo de Alimentos Ultraprocessados: Descrição e Avaliação de Desempenho No Brasil. Rev. Saude Publ..

[96] Franca P.A.P., Duque-Estrada P., da Fonseca e Sá B.F., van der Goot A.J., Pierucci A.P.T.R. (2022). Meat Substitutes—Past, Present, and Future of Products Available in Brazil: Changes in the Nutritional Profile. Future Foods.

[97] Nolden A.A., Forde C.G. (2023). The Nutritional Quality of Plant-Based Foods. Sustainability.

[98] Reyneke G., Hughes J., Grafenauer S. (2022). Consumer Understanding of the Australian Dietary Guidelines: Recommendations for Legumes and Whole Grains. Nutrients.

[99] Vanga S.K., Raghavan V. (2018). How Well Do Plant Based Alternatives Fare Nutritionally Compared to Cow’s Milk?. J. Food Sci. Technol..

[100] Appleton K.M. (2023). Barriers to and Facilitators of the Consumption of Animal-Based Protein-Rich Foods in Older Adults: Re-Analysis with a Focus on Sustainability. Nutrients.

[101] Cardello A.V., Llobell F., Giacalone D., Roigard C.M., Jaeger S.R. (2022). Plant-Based Alternatives vs Dairy Milk: Consumer Segments and Their Sensory, Emotional, Cognitive and Situational Use Responses to Tasted Products. Food Qual Prefer..

[102] Ciobanu M., Manoliu D., Anchidin B. (2023). The Influence of Sensory Characteristics of Game Meat on Consumer Neuroperception: A Narrative Review. Foods.

[103] Derbyshire E. (2022). Food-Based Dietary Guidelines and Protein Quality Definitions—Time to Move Forward and Encompass Mycoprotein?. Foods.

[104] Jagdale Y.D., Mahale S.V., Zohra B., Nayik G.A., Dar A.H., Ali Khan K., Abdi G., Karabagias I.K. (2021). Nutritional Profile and Potential Health Benefits of Super Foods: A Review. Sustainability.

[105] Meyerding S.G.H. (2016). Consumer Preferences for Food Labels on Tomatoes in Germany—A Comparison of a Quasi-Experiment and Two Stated Preference Approaches. Appetite.

[106] Qian F., Riddle M.C., Wylie-Rosett J., Hu F.B. (2020). Red and Processed Meats and Health Risks: How Strong Is the Evidence?. Diabetes Care.

[107] Schmid A. (2011). The Role of Meat Fat in the Human Diet. Crit. Rev. Food Sci. Nutr..

[108] Golzarand M., Bahadoran Z., Mirmiran P., Azizi F. (2016). Protein Foods Group and 3-Year Incidence of Hypertension: A Prospective Study From Tehran Lipid and Glucose Study. J. Ren. Nutr..

[109] Nelson D.L., Cox M.M. (2000). Lehninger Principles of Biochemistry.

[110] Szenderák J., Fróna D., Rákos M. (2022). Consumer Acceptance of Plant-Based Meat Substitutes: A Narrative Review. Foods.

[111] Freeland-Graves J.H., Peckam G.C. (1987). Foundations of Food Preparation.

[112] Bleiweiss-Sande R., Chui K., Evans E.W., Goldberg J., Amin S., Sacheck J. (2019). Robustness of Food Processing Classification Systems. Nutrients.

[113] Poti J.M., Mendez M.A., Ng S.W., Popkin B.M. (2015). Is the Degree of Food Processing and Convenience Linked with the Nutritional Quality of Foods Purchased by US Households ?. Am. J. Clin. Nutr..

[114] Rabassa M., Hernández Ponce Y., Garcia-Ribera S., Johnston B.C., Salvador Castell G., Manera M., Pérez Rodrigo C., Aranceta-Bartrina J., Martínez-González M.Á., Alonso-Coello P. (2022). Food-Based Dietary Guidelines in Spain: An Assessment of Their Methodological Quality. Eur. J. Clin. Nutr..

[115] Steele E.M., Baraldi L.G., Da Costa Louzada M.L., Moubarac J.C., Mozaffarian D., Monteiro C.A. (2016). Ultra-Processed Foods and Added Sugars in the US Diet: Evidence from a Nationally Representative Cross-Sectional Study. BMJ Open.

[116] Slavin J.L., Lloyd B. (2012). Health Benefits of Fruits and Vegetables. Am. Soc. Nutr..

[117] Zhu F., Du B., Xu B. (2018). Anti-Inflammatory Effects of Phytochemicals from Fruits, Vegetables, and Food Legumes: A Review. Crit. Rev. Food Sci. Nutr..

[118] Imamura F., O’Connor L., Ye Z., Mursu J., Hayashino Y., Bhupathiraju S.N., Forouhi N.G. (2015). Consumption of Sugar Sweetened Beverages, Artificially Sweetened Beverages, and Fruit Juice and Incidence of Type 2 Diabetes: Systematic Review, Meta-Analysis, and Estimation of Population Attributable Fraction. BMJ.

[119] Auerbach B.J., Wolf F.M., Hikida A., Vallila-Buchman P., Littman A., Thompson D., Louden D., Taber D.R., Krieger J. (2017). Fruit Juice and Change in BMI: A Meta-Analysis. Pediatrics.

[120] Crowe-White K., O’Neil C.E., Parrott J.S., Benson-Davies S., Droke E., Gutschall M., Stote K.S., Wolfram T., Ziegler P. (2016). Impact of 100% Fruit Juice Consumption on Diet and Weight Status of Children: An Evidence-Based Review. Crit. Rev. Food Sci. Nutr..

[121] Cámara M., Giner R.M., González-Fandos E., López-García E., Mañes J., Portillo M.P., Rafecas M., Domínguez L., Martínez J.A. (2021). Food-Based Dietary Guidelines around the World: A Comparative Analysis to Update Aesan Scientific Committee Dietary Recommendations. Nutrients.

[122] Deshpande S.S. (1992). Food Legumes in Human Nutrition: A Personal Perspective. Crit. Rev. Food Sci. Nutr. Food Legumes Hum. Nutr..

[123] Nwokolo E., Smartt J. (1996). Food and Feed from Legumes and Oilseeds.

[124] USDA Food Composition Databases. https://ndb.nal.usda.gov/ndb/foods.

[125] Kristensen M.D., Bendsen N.T., Christensen S.M., Astrup A., Raben A. (2016). Meals Based on Vegetable Protein Sources (Beans and Peas) Are More Satiating than Meals Based on Animal Protein Sources (Veal and Pork)—A Randomized Cross-over Meal Test Study. Food Nutr. Res..

[126] Paul A.A., Kumar S., Kumar V., Sharma R. (2020). Milk Analog: Plant Based Alternatives to Conventional Milk, Production, Potential and Health Concerns. Crit. Rev. Food Sci. Nutr..

[127] Chaves L.H.G., Francisco P.R.M., de Vasconcelos A.C.F. (2019). Oleaginosas e Hortaliças Sob Diferentes Manejos de Cultivo: Coletânea de Estudos.

[128] Dibakoane S.R., Du Plessis B., Da Silva L.S., Anyasi T.A., Emmambux M.N., Mlambo V., Wokadala O.C. (2022). Nutraceutical Properties of Unripe Banana Flour Resistant Starch: A Review. Starch.

[129] Falcomer A.L., Riquette R.F.R., De Lima B.R., Ginani V.C., Zandonadi R.P. (2019). Health Benefits of Green Banana Consumption: A Systematic Review. Nutrients.

[130] Van Huis A. (2013). Potential of Insects as Food and Feed in Assuring Food Security. Annu. Rev. Entomol..

[131] Nowak V., Persijn D., Rittenschober D., Charrondiere U.R. (2016). Review of Food Composition Data for Edible Insects. Food Chem..

[132] Zambiazi R.C., Przybylski R., Zambiazi M.W., Mendonça C.B. (2007). Fatty Acid Composition of Vegetable Oils and Fats. Bol. Cent. Pesqui. Process. Aliment..

[133] Burlingame B., Nishida C., Uauy R., Weisell R. (2009). Fats and Fatty Acids in Human Nutrition: Introduction. Ann. Nutr. Metab..

[134] Gomna A., N’Tsoukpoe K.E., Le Pierrès N., Coulibaly Y. (2019). Review of Vegetable Oils Behaviour at High Temperature for Solar Plants: Stability, Properties and Current Applications. Sol. Energy Mater. Sol. Cells.

[135] Marriott B.P., Olsho L., Hadden L., Connor P. (2010). Intake of Added Sugars and Selected Nutrients in the United States, National Health and Nutrition Examination Survey (NHANES) 2003–2006. Crit. Rev. Food Sci. Nutr..

[136] Archer E., Arjmandi B. (2021). Falsehoods and Facts about Dietary Sugars: A Call for Evidence-Based Policy. Crit. Rev. Food Sci. Nutr..

[137] Monteiro C.A., Levy R.B., Claro R.M., de Castro I.R.R., Geoffrey C. (2010). A New Classification of Foods Based on the Extent and Purpose of Their Processing Uma Nova Classifi Cação de Alimentos Baseada Na Extensão e Propósito Do Seu Processamento. Cad Saude Publica.

[138] Amorim A., Laurindo J., Sobral P.J. (2022). On How People Deal with Industrialized and Non-Industrialized Food: A Theoretical Analysis. Front. Nutr..

[139] Guiné R.P.F., Florença S.G., Carpes S., Anjos O. (2020). Study of the Influence of Sociodemographic and Lifestyle Factors on Consumption of Dairy Products: Preliminary Study in Portugal and Brazil. Foods.

[140] Anastasiou K., Miller M., Dickinson K. (2019). The Relationship between Food Label Use and Dietary Intake in Adults: A Systematic Review. Appetite.

[141] Brown K.A., Timotijevic L., Barnett J., Shepherd R., Lähteenmäki L., Raats M.M. (2011). A Review of Consumer Awareness, Understanding and Use of Food-Based Dietary Guidelines. Br. J. Nutr..

[142] Van der Merwe D., Bosman M., Ellis S. (2014). Consumers’ Opinions and Use of Food Labels: Results from an Urban–rural Hybrid Area in South Africa. Food Res. Int..

[143] Guzik P., Szymkowiak A., Kulawik P., Zając M. (2022). Consumer Attitudes towards Food Preservation Methods. Foods.

[144] McCluskey J.J., Wesseler J., Winfree J.A. (2018). The Economics and Politics GM Food Labeling: An Introduction to the Special Issue. Food Policy.

[145] Peretti A.P.d.R., Araujo W.M.C. (2010). Scope of Safety Requirement in Quality Certificates Used in Food Production in Brazil. Gestão Produção.

[146] Souza A.M., Bezerra I.W.L., Pereira G.S., Torres K.G., Costa R.M., Oliveira A.G. (2020). Relationships between Motivations for Food Choices and Consumption of Food Groups: A Prospective Cross-Sectional Survey in Manufacturing Workers in Brazil. Nutrients.

[147] Sichieri R., Chiuve S.E., Rosângela A.P., Aline Cristine S.L., Willett W.C. (2010). Dietary Recommendations: Comparing Dietary Guidelines from Brazil and the United States Recomendações Dietéticas: Comparação Entre Os Guias Alimentares Brasileiro e Americano. Cad Saude Publica.

[148] De Andrade L.M., Bocca C. (2016). Análise Comparativa de Guias Alimentares: Proximidades e Distinções Entre Três Países The Comparative Analysis of Dietary Guidelines: Similarities and Distinctions between Three Countries. Demetra.

[149] Ireland J.D., Møller A. (2016). Food Classification and Description. Encyclopedia of Food and Health.

[150] Jomori M.M., da Costa Proença R.P., Calvo M.C.M. (2008). Determinantes de Escolha Alimentar. Rev. Nutr..

[151] Jomori M.M., da Costa Proença R.P., Calvo M.C.M. (2008). Escolha Alimentar: A Questão de Género No Contexto Da Alimentação Fora de Casa. Cad. Espaço Fem..

[152] Deharveng G., Charrondie U.R., Slimani N., Southgate D.A.T., Riboli E. (1999). Comparison of Nutrients in the Food Composition Tables Available in the Nine European Countries Participating in EPIC. Eur. J. Clin. Nutr..

[153] Charrondière R.U., Stadlmayr B., Rittenschober D., Mouille B., Nilsson E., Medhammar E., Olango T., Eisenwagen S., Persijn D., Ebanks K. (2013). FAO/INFOODS Food Composition Database for Biodiversity. Food Chem..

[154] Scrimshaw N.S. (1997). INFOODS: The International Network of Food Data Systems. Am. J. Clin. Nutr..

[155] Menegassi B., de Morais Sato P., Scagliusi F.B., Moubarac J.-C. (2019). Comparing the Ways a Sample of Brazilian Adults Classify Food with the NOVA Food Classification: An Exploratory Insight. Appetite.

[156] Navas S., Trombini A., Rebecchi P. (1996). DAFNE Program Description (No. DELPHI-96-46-PROG-215). https://cds.cern.ch/record/2627773/files/96_46_prog_215.pdf.

[157] Brussaard J.H., Löwik M.R.H., Steingrimsdottir L., Møller A., Kearney J., De Henauw S., Becker W. (2002). A European food consumption survey method–conclusions and recommendations. Eur. J. Clin. Nutr..

[158] PAHO/WHO (2016). Pan American Health Organization. Pan American Health Organization Nutrient Profile Model. https://iris.paho.org/bitstream/handle/10665.2/18621/9789275118733_eng.pdf.

